
JOURNAL INFORMATION
==============================
NLM Title Abbreviation: Ann R Coll Surg Engl
Journal ID: Ann R Coll Surg Engl
NLM Journal ID: 7506860
Journal ID: ann

Annals of The Royal College of Surgeons of England

ISSN: 0035-8843
EISSN: 1478-7083
Publisher: Royal College of Surgeons of England

ARTICLE INFORMATION
==============================
PMCID: PMC10929724
DOI: 10.1308/rcsann.2024.0025
Article ID: rcsann.2024.0025
Article version: 1
Subjects: Oral Presentations

Oral Presentations

Print publication date: 2024 Mar
Volume: 106
Issue: Suppl 1
First page: S1
Last page: S2
Copyright: Copyright © 2024, The Authors
Copyright year: 2024
License: This is an open-access article distributed under the terms of the Creative Commons Attribution 4.0 International License, which permits unrestricted use, distribution, reproduction, and adaptation in any medium, provided the original work is properly attributed.
License URL: https://creativecommons.org/licenses/by/4.0/

==============================

Subjects: Oral Presentations

256 Advancing hip dysplasia detection in adults: a convolutional neural network approach

Yasen Z 1
Logishetty K 2
Edwards T 1
1 Imperial College London , London, UK
2 Imperial College University , London, UK

15 From bones to benches: 3D printed cortico-medullary differentiated metacarpal simulators: the next step in sustainable hand fracture fixation training

Gundle L 1
Moffat M 2
Owens A 2
Gregson R 2
1 Royal Free Hospital , London, UK
2 University of Bristol , Bristol, UK

56 Welsh Centre for Burns and Plastic Surgery trial of immersive virtual reality as distraction in paediatric patients

Jones E 1
Edwards K 1
Breed H 2
Cubitt J 1
1 Morriston Hospital , Swansea, UK
2 Buckingham NHS Trust , Buckingham, UK

169 Use of virtual reality in the education of orthopaedic procedures: a randomised control study in early validation of a novel virtual reality simulator

Sehdev S 1 2
Gomindes A 1
Adeeko ES 1 2
Khatri C 1 2
Carlos J 1 2
Ahmed I 1 2
Ward T 1 2
Leverington J 1 2
Debenham L 1 2
Metcalfe A 1 2
Ward J 1 2
1 University Hospital Coventry and Warwickshire , Coventry, UK
2 University of Warwick , Warwick, UK

202 A simple technique using a Venflon to fix fractures of the glenoid

Kapasi A
Uzoigwe C
Barlow D
McMurtrie A
Wrexham Maelor Hospital , Wrexham, UK

